# The UK Biobank imaging enhancement of 100,000 participants: rationale, data collection, management and future directions

**DOI:** 10.1038/s41467-020-15948-9

**Published:** 2020-05-26

**Authors:** Thomas J. Littlejohns, Jo Holliday, Lorna M. Gibson, Steve Garratt, Niels Oesingmann, Fidel Alfaro-Almagro, Jimmy D. Bell, Chris Boultwood, Rory Collins, Megan C. Conroy, Nicola Crabtree, Nicola Doherty, Alejandro F. Frangi, Nicholas C. Harvey, Paul Leeson, Karla L. Miller, Stefan Neubauer, Steffen E. Petersen, Jonathan Sellors, Simon Sheard, Stephen M. Smith, Cathie L. M. Sudlow, Paul M. Matthews, Naomi E. Allen

**Affiliations:** 1 Nuffield Department of Population Health, University of Oxford, Oxford, UK; 2 Usher Institute of Population Health Sciences and Informatics, University of Edinburgh, Edinburgh, UK; 3 Department of Clinical Radiology, New Royal Infirmary of Edinburgh, Edinburgh, UK; 4 UK Biobank Coordinating Centre, Stockport, UK; 5 Centre for Functional MRI of the Brain, Wellcome Centre for Integrative Neuroimaging, University of Oxford, Oxford, UK; 6 Research Centre for Optimal Health, University of Westminster, London, UK; 7 Birmingham Women’s and Children’s NHS Foundation Trust, Birmingham, UK; 8 Department of Cardiovascular Sciences and Electrical Engineering, KU Leuven, Leuven, Belgium; 9 CISTIB Centre for Computational Imaging and Simulation Technologies in Biomedicine, Schools of Computing and Medicine, University of Leeds, Leeds, UK; 10 MRC Lifecourse Epidemiology Unit, University of Southampton, Southampton, UK; 11 Radcliffe Department of Medicine, University of Oxford, Oxford, UK; 12 William Harvey Research Institute, Queen Mary University of Medicine, London, UK; 13 Department of Brain Sciences, Imperial College London and UK Dementia Research Institute, London, UK

**Keywords:** Imaging, Medical research

## Abstract

UK Biobank is a population-based cohort of half a million participants aged 40–69 years recruited between 2006 and 2010. In 2014, UK Biobank started the world’s largest multi-modal imaging study, with the aim of re-inviting 100,000 participants to undergo brain, cardiac and abdominal magnetic resonance imaging, dual-energy X-ray absorptiometry and carotid ultrasound. The combination of large-scale multi-modal imaging with extensive phenotypic and genetic data offers an unprecedented resource for scientists to conduct health-related research. This article provides an in-depth overview of the imaging enhancement, including the data collected, how it is managed and processed, and future directions.

## Abstract (web summary)

Between 2014 and 2023, 100,000 UK Biobank participants are undergoing brain, heart and abdominal MRI, as well as DXA and carotid ultrasound scans. In this review, authors provide a detailed overview of the rationale for the collection of these imaging data, the procedures of data collection and management, and the future directions of the UK biobank imaging enhancement.

## Introduction

Imaging provides structural and functional information on internal anatomy and physiological processes. Its use in clinical practice has transformed the diagnosis, management and treatment of disease. Imaging can detect asymptomatic pathology before disease development and thus can be used to screen high-risk populations to support precision and preventative medicine. In some cases, imaging can provide insights into the biological mechanisms underlying exposure-disease associations.

Large-scale population-based prospective studies can facilitate the identification of imaging measures as targets for prevention or provide an insight into disease mechanisms. Although some epidemiological studies have incorporated imaging measures, these have usually been limited to a specific imaging modality or body region (such as the brain or heart), have often been restricted to selective population subgroups at high risk for certain diseases and have included no more than a few thousand participants. For example, the first cohort studies to use magnetic resonance imaging (MRI), such as the Rotterdam study and the Multi-Ethnic Study of Atherosclerosis (MESA), included <5000 participants^1,2^.

However, to assess the moderate associations that may exist between genetic and lifestyle factors and imaging-derived phenotypes (IDPs), or between IDPs and subsequent risk of a wide range of diseases, it is necessary to perform imaging in very large numbers of healthy individuals as only a relatively small proportion of them will develop any particular condition during follow-up. Furthermore, in the era of ‘Big Data’, large, diversely phenotyped cohorts are essential to maximise recent developments in artificial intelligence (AI). To address this challenge, more ambitious multi-modal imaging protocols have been initiated in longitudinal cohorts, including brain and body MRI in the German National Cohort on 30,000 participants^3^, and MRI of the brain, blood vessels, heart and liver in the Canadian Partnership for Tomorrow Project (CPTP) for 10,000 participants (see www.partnershipfortomorrow.ca).

Here, we provide an overview of the programme currently underway in UK Biobank (UKB), the largest and most detailed imaging study to date. The UKB imaging enhancement aims to perform brain, cardiac and abdominal MRI, full body dual-energy X-ray absorptiometry (DXA) and a carotid ultrasound scan on 100,000 of the existing 500,000 UKB participants before the end of 2023^4^. As of early 2020, over 45,000 participants have undergone an assessment, already making the UKB imaging enhancement by far the largest multi-modal imaging study in the world. This article outlines the scientific rationale and processes involved in collecting, curating and disseminating the imaging data for research purposes, and describes recent developments, such as the initiative to repeat the imaging of at least 10,000 participants.

## The launch of UK Biobank

Between 2006 and 2010, 9.2 million women and men aged 40–69 who were registered with the UK’s National Health Service (NHS) were sent postal invitations to attend one of 22 UKB assessment centres in England, Scotland and Wales^5^. Of these, ~500,000 (5.5%) individuals joined the study. Although UKB is not representative of the UK population, the large sample size and heterogeneity of measures nonetheless enable a valid assessment of many exposure–outcome relationships to be made. During the baseline assessment, extensive sociodemographic, lifestyle and health-related information was collected through a touchscreen questionnaire and verbal interview, and a wide range of physical measures were performed^4,6^. Participants also provided biological samples that have been used to perform genotyping^7^ and haematological and biochemistry assays for the full cohort^8^. Once recruitment was fully underway, additional measures were incorporated into the baseline assessment, including tests of hearing and arterial stiffness (*n* = ~200,000), a cardiorespiratory fitness test (*n* = ~100,000) and various eye measures (*n* = 100,000–150,000). Since the baseline visit, subsets of participants have supported additional data collection through various enhancements to the study. These have included: a full repeat of the baseline assessment (*n* = ~20,000, 2012–2013), collection of physical activity data over 7-days by wearing accelerometers (*n* = ~100,000, 2013–2015 and *n* = ~2500 on four occasions, 2018–2019) and regular online questionnaires covering a variety of topics such as diet, cognitive function, occupational history, mental wellbeing, gastrointestinal health and pain (sent to ~330,000 participants with email addresses; ~35–50% response rate for each questionnaire).

All participants provided consent for their health to be followed-up through linkage to health-related records, which currently includes death, cancer and hospital inpatient records for the full cohort. Primary care data are also available for ~45% of the cohort (with data for the remaining participants pending). Together, these electronic medical record data capture information on type and date of diagnosis and symptoms, procedures and operations, prescriptions, test results and referrals by general practitioners.

UKB received approval from the National Information Governance Board for Health and Social Care and the National Health Service North West Centre for Research Ethics Committee (Ref: 11/NW/0382). UKB is compliant with both the previous Data Protection Act and the more recent General Data Protection Regulation (GDPR) implemented in 2018. For the GDPR, participants were contacted by email or post to explain how UKB meets the requirements of the new regulations (https://www.ukbiobank.ac.uk/gdpr/).

### Rationale for multi-modal imaging on 100,000 participants in UK Biobank

When the original UKB protocol was reviewed in 2006, the UK Biobank International Peer Review Panel recommended exploring the feasibility of conducting enhancements in large subsets of the cohort. The inclusion of imaging measures was deemed of value and further consultation with the wider scientific community was recommended. Consequently, UKB established an expert Imaging Working Group in 2011 who, after consultation with over 100 imaging specialists worldwide, developed an imaging protocol that aimed to maximise the scientific value of the imaging data collected while also being achievable at scale (i.e., non-invasive with short acquisition times).

A key requirement was the inclusion of several imaging modalities that could provide precise and reliable information on multiple organ systems as opposed to single body sites. The protocol thus includes collection of imaging data on the brain, heart, large blood vessels, body composition, bone and joints. This provides researchers with the opportunity to use measures from different organ systems to explore the multifactorial biological mechanisms of complex diseases. For example, the diversity of data could contribute to a better understanding of the relationships between systemic health and dementia through capture of data regarding the structure and function of the brain (brain MRI) in conjunction with adiposity (body MRI and DXA) and vascular risk factors (cardiac MRI and carotid ultrasound)^9^. The integration of multiple ‘gold-standard’ imaging measures can also be used to calibrate and expand upon the data collected at the baseline assessment. For example, the whole-body DXA scan complements measures of bone density obtained from the heel ultrasound performed at baseline. Further, body MRI and DXA could provide more detailed data on body composition and fat distribution than that provided by the bioimpedance measures. This will support more refined analyses of body composition, such as whether disease risk varies in those with a normal body mass index (BMI) but who have a high visceral fat content^10^.

Another consideration for the Imaging Working Group was how many participants to image. This was done by ultimately balancing estimates of the power of potential future nested case-control studies with pragmatic considerations based on costs and feasibility. Approximately 5000 and 10,000 cases are required to detect an odds ratio of 1.5 and 1.33 with 80% power, respectively, when the exposure prevalence is 10%^11^. Although imaging 100,000 participants is unprecedented, it is clear this is the sample size needed to capture sufficient cases to reliably explore a wide range of associations.

### Protocols of the UK Biobank imaging enhancement

Following ethical approval, a pilot study of ~5,000 participants was performed between 2014 and 2015 to demonstrate the feasibility of high-throughput imaging and to finalise the imaging protocols required for the main phase. Funding was then released to extend the imaging enhancement to an additional 95,000 participants, with data collection estimated to finish by 2023. The imaging assessment takes place in dedicated, purpose-built centres based in Stockport (termed Central), Newcastle-upon-Tyne (North), Reading (South-East) and Bristol (South-West). The locations were selected to minimise travel times for the majority of participants, based on driving times and availability of public transport links, as travelling time was recognised as one of the main determinants as to whether a participant was likely to attend or not.

### Invitation process

Invitations for the Central region of the UK began in April 2014, followed by the Northern region in April 2017, the South-East region in June 2018 and the South-West centre in February 2020. Initially, invitations were sent by email as this is the most cost-effective means of communicating with participants. However, as not all participants provided an email address, postal invitations began in early 2020 to provide all participants the opportunity to attend, should they wish to do so. Therefore, all surviving UKB participants will be invited, except for those who have informed UKB they no longer wish to be contacted or now live outside the UK (<0.5% of participants to date). Participants are provided with comprehensive information about the project, including an invitation letter briefly describing what the assessment visit involves and an information leaflet describing the individual scans, eligibility criteria and benefits and risks of participation as well as links to a dedicated UKB imaging website (https://imaging.ukbiobank.ac.uk/). If interested, participants are asked to telephone the Participant Resource Centre who makes an initial assessment as to whether the potential participants are eligible for inclusion in the enhancement (e.g., the MRI scans are not safe for individuals who have metal implants or who have had certain surgeries) and for tolerability (e.g., claustrophobia). Completion of the full protocol is not possible for those unable to lie still, hold their breath voluntarily or hear instructions. Participants who have MR-compatible metal implants in their body (not limbs) are also excluded as these can affect the quality of the scan in regions close to the metal and reduce their value for research purposes.

**Fig. 1:**
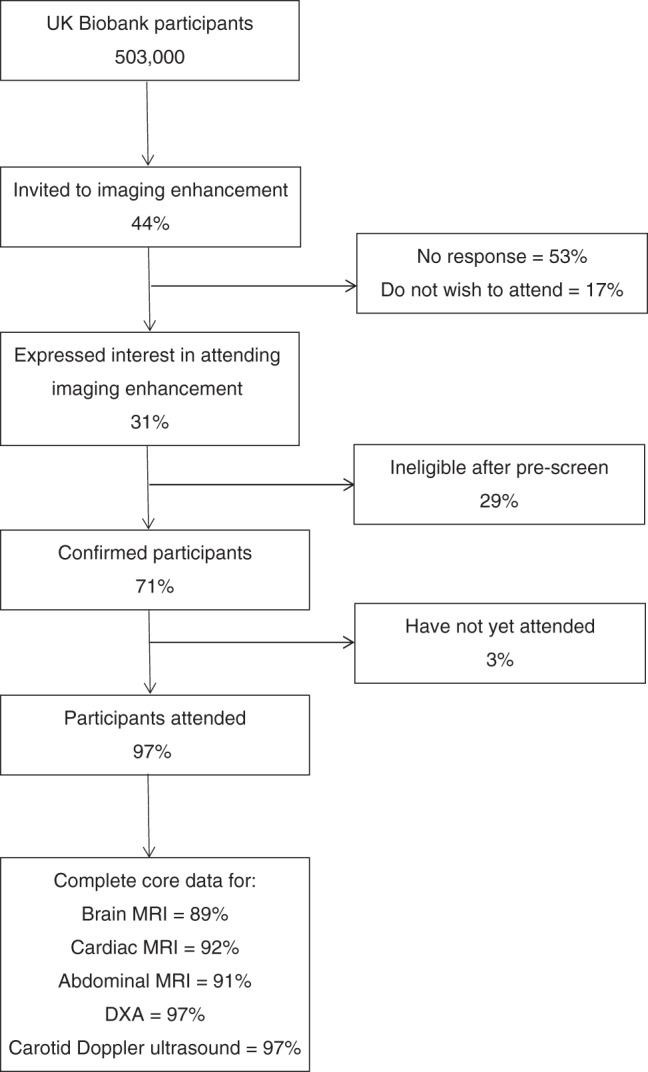
Flow chart of participation in the UK Biobank multi-modal imaging study. Note that as the invitation process is ongoing, this flow chart is only accurate as of early 2020. For example, some participants classified as ‘no response’ might attend the imaging enhancement in future.

Reminder emails are sent to non-responders 2 weeks, 4 weeks, 6 months, 12 months and 24 months after the initial invitation. All email invitations and reminders contain a decline link, where participants can opt out of receiving subsequent invitations. To obtain imaging data on 100,000 participants, an attendance rate of at least 20% of the 500,000 UKB participants is required. To date, this has been achieved, with 31% of invited participants expressing an interest, of which 71% are eligible and have booked an appointment; of these, 97% have attended an imaging assessment centre (Fig. 1). Approximately 12% of participants book an appointment in response to an initial invitation, with response rates of 7% after the 2 week reminder and a further 4% after the 4-week reminder.

### General imaging process

The target throughput for each imaging centre is 18 participants per 12-hour working day, and the centres are open every day (except for the Christmas and Easter holidays). When fully operational, a monthly average of 17 participants per day is achieved (with 95% attendance rate). Each centre is staffed by six radiographers, three healthcare assistants, a laboratory specialist, a healthcare assistant team leader and a centre manager, with a lead radiographer and MR physicist providing support across all centres. Four sub-specialist third party consultant radiologists, each with experience in brain, cardiac, abdominal or musculoskeletal imaging, review scans that are flagged by radiographers as having a potentially serious incidental finding.

On arrival at the assessment centre, the participant’s eligibility is again checked with a radiographer, and electronic consent is obtained to confirm that the participant understands the nature of the study and potential implications, such as the detection and feedback of incidental findings. There are four imaging stations: one consisting of the brain MRI, another one covering both the cardiac and abdominal MRI, one for DXA and a final station for carotid ultrasound. To fully maximise the use of the facilities, three participants go to a separate station simultaneously and serially rotate through different stations. Once participants have finished the imaging assessments, they repeat all the measures collected at the baseline assessment, except for the eye examinations, 4-lead electrocardiogram (ECG) during exercise and saliva sampling.

### General imaging quality control

A centralised training and monitoring team is responsible for quality assurance across all imaging centres. All staff members undergo a six-week training programme before centres open, with monthly training provided by the MR physicist. To ensure fully harmonised imaging data are acquired across centres, identical scanner models, software, adjustment and tuning methods, types of coils and protocols are used. Quality assurance and control measures are also in place including a standardised training programme for all radiographers in each centre, system acceptance testing, standard operating procedures, as well as routine phantom measurements, regular servicing and performance checks that are conducted by a dedicated UKB physicist. The radiographer visually inspects the MRI images for quality control purposes while participants are undergoing scanning and immediately after acquisition. Quality control assessments consisting of qualitative and quantitative comparisons conducted by external imaging experts for each modality confirmed that images acquired during the pilot study were of excellent quality for research purposes (Table 1).

**Table 1:** Quality control performed on brain, cardiac and abdominal MRI, DXA and carotid ultrasound sequences/images during the pilot study.

Imaging modality	Assessor	Sequence/images assessed	Number images assessed	Quality control metrics	Passed quality control (%)
Brain MRI	FMRIB	T1	2957	Similarity to template after non-linear registration (alignment)	99
				Signal to noise	99
				Contrast to noise	99
		T2/Flair	2957	Similarity to T1 after linear registration	99
		T2^*^ (magnitude image)	2957	Similarity to T1 after linear registration	100
		Resting fMRI	2957	Similarity to T1 after linear registration	100
				Temporal signal to noise	99
				High subject head motion	96
		Task fMRI	2957	Similarity to T1 after linear registration	100
				Temporal signal to noise	98
				High subject head motion	96
		Diffusion MRI	2957	Similarity to T1 after linear registration	100
	BioMedIA	T1	100	Whole brain tissue segmentation	100
	INRIA Asclepios	T1	100	Cortical segmentation	100
Cardiac MRI	BioMedIA	Short-axis, cine views	100	Segmentation of myocardium and ventricular blood pools	100
	INRIA Asclepios	Short-axis cine views	100	Visual inspection	85
				Automated displacement and strain analyses	100
	QMUL	All images	100	Manual “reference” analysis of all proposed IDPs feasible, excellent intra-observer variability of all IDPs	100
	Oxford	ShMOLLI TI map	100	TI mapping	90
	Yale	Short-axis, cine views	100	Visual inspection, processing pipeline works	100
	Leeds CISTIB/LICAMM	Short-axis, cine views	100	Visual assessment, segmentation of myocardium and blood pools, image registration	100
	Auckland	Automatic in line LV function	100	Comparison to manual analysis	99
	Oxford UKB cluster	Automatic in line LV function	3456	Automated extraction of LV measures	99
Abdominal MRI	BioMedIA	Dixon whole body	100	“Stitching” 6 scan stations for whole body reconstruction (24 scans per subject)	93
	Prof. Jimmy Bell	T1	100	Visual	96
		T2^*^	100	Visual	95
		Dixon	100	Visual	96
	AMRA	Dixon abdomen station images	100	Semi-automated tissue segmentation	99.7
		Dixon thigh station images	100	Semi-automated multi-atlas tissue segmentation	98.1
	Perspectum	Liver	100	Semi-automated fibrosis index	96
			100	Semi-automated steatosis measure	99
			100	Semi-automated haemosiderosis measure	99
DXA	Dr Nicola Crabtree	DXA	1837	Visual comparison versus gold standard	99
				Quantitative comparison of all measures versus those re-derived by expert	99
	GE iDXA device	DXA	3222	Total and region-specific bone mineral density measures	99–100
				Total and region-specific body composition measures	96–100
Carotid ultrasound	UKB senior radiographer and Prof. Paul Leeson	Right and left carotid images	1994	Visual comparison versus expert gold standard	99
	Panasonic imaging device	Right and left carotid images	3107	Right and left carotid intima-media thickness	96–99^a^

*DXA* dual-energy X-ray absorptiometry, *fMRI* functional magnetic resonance imaging, *IDP* imaging-derived phenotype, *LV* left ventricle, *MRI* magnetic resonance imaging, *UKB* UK Biobank.

^a^96% ≥2 sets of measures obtained from both left and right carotids; 99% ≥1 set of measures obtained from left or right carotid.

### Incidental findings

UKB developed an approach to managing the clinical review of images acquired in consultation with stakeholders, funders and the UKB Ethics and Governance Council (now the Ethics Advisory Committee). Consistent with the practices established for other data collected by UKB, participants are informed that the data collected is intended for research use only, that the scans will not be routinely assessed for evidence of disease and that individual results will not be made available to them (detailed information on incidental findings can be found at https://imaging.ukbiobank.ac.uk). However, and consistent with the approach adopted for incidental findings during the original assessment visit, if while scanning a participant, a radiographer observes an incidental finding that might be clinically serious or life-threatening then the relevant scans undergo further review by a specialist radiologist, who determines independently whether UKB should notify the participant and their general practitioner. All participants explicitly consent to participate in the imaging enhancement on this basis.

This approach was evaluated through comparison with systematic radiology review of all images from the first 1000 imaged participants. Compared to the systematic radiologist review, radiographer flagging resulted in substantially fewer participants with potentially serious incidental findings (179/1000 [17.9%] versus 18/1000 [1.8%]) but a higher proportion with serious final diagnoses (21/179 [11.7%] versus 5/18 [27.8%]). Radiographer flagging missed 16/21 serious final diagnoses (false negatives) while systematic radiologist review generated large numbers of non-serious final diagnoses (158/179, false positives). All participants who were notified of a potentially serious incidental finding consulted their GP, and 90% had some further clinical assessment (most commonly additional imaging (79%), or referral to a specialist (64%)). Some participants reported that feedback of their incidental findings had a negative impact on their emotional wellbeing, insurance status or finances, or work and leisure activities (17%, 9% and 6%, respectively).

In light of these findings from the pilot and with additional advice from UKB’s independent Ethics and Governance Council, we concluded that the proposed UKB imaging incidental findings protocol to use radiographer flagging (and not systematic radiologist review) provides an acceptable balance of benefit versus harm to the participants, as detailed elsewhere^12^.

### Rationale, protocol and data processing for each imaging modality Brain MRI

There are several neuroimaging methods that can measure different aspects of the brain. However, MRI is unique as it can capture high-resolution structural and functional information in a single examination in a non-invasive manner (i.e., with no use of non-ionising radiation). Both structural and functional brain measures show promise as markers to guide strategies for disease prevention, monitoring of disease progression or as predictive markers for disease risk (e.g., by identifying neuroanatomical markers related to the risk of developing dementia)^13^. However, although brain MRI has been used commonly for smaller clinical and non-clinical neuroscientific research^14^, its use in large-scale population-based epidemiological studies like UKB is limited.

The brain MRI protocol is performed using a 3 Tesla Siemens Skyra scanner (Siemens Healthineers, Erlangen, Germany) with VD13 software and a 32-channel head coil. The full examination lasts approximately 35 min. Table 2 reports the selected parameters of the brain MRI protocols. The protocol includes three structural MRI scans; T1, T2 fluid attenuation inversion recovery (FLAIR) and susceptibility-weighted MRI (swMRI), as well as diffusion MRI (dMRI) and resting and task functional MRI (fMRI). T1 scans allow precise volumetric measures of the whole brain, as well as specific cortical and subcortical regions. The T2 FLAIR scan identifies changes that might be indicative of inflammation or tissue damage. For instance. an increased signal in the white matter is associated with an increased risk of dementia and stroke^15^. swMRI is sensitive to increased iron content as a result of microbleeds or chronic microglial activation in the context of neurodegeneration^16^. dMRI reflects structural connectivity and tissue microstructural features describing white matter integrity. Resting fMRI is performed on an individual who is not engaged in any particular activity or task and can provide indices related to the functional connectivity between brain regions independent of external stimuli. By contrast, task fMRI is performed on an individual to whom stimuli are repetitively delivered that engage sensory-motor and cognitive processes of interest. The UKB task fMRI protocol is based on the Hariri faces/shapes ‘emotion’ task, selected because it engages a wide range of cognitive and sensory-motor systems and a wide range of normative data is available^17^.

**Table 2:** Brain MRI protocol parameters.

Modality	Duration (mins)	Resolution (mm^3^)	Matrix	Other parameters
T1 MPRAGE	4:54	1.0x1.0x1.0	256x256x208	TI/TR = 880/2000 ms, R = 2
Resting fMRI	6:10	2.4x2.4x2.4	88x88x64	TE/TR = 39/735 ms, α = 51°, MB = 8
T2 FLAIR	5:52	1.0x1.0x1.05	256x256x192	TI/TR = 1800/5000 ms, R = 2
Diffusion MRI^1^	7:08	2.0x2.0x2.0	104x104x72	TR = 3600 ms, 50 directions/shell, b = 0, 1000, 2000 s/mm2, α = 51°, MB = 3
Susceptibility-weighted	2:34	0.8x0.8x3.0	288x256x48	TE1/TE2/TR = 9.4/20/27 ms, R = 2
Task fMRI	4:13	2.4x2.4x2.4	88x88x64	TE/TR = 39/735 ms, α = 51°, MB = 8

FLAIR, fluid-attenuated inversion recovery; MB, multi-band factors; MPRAGE, magnetization-prepared rapid acquisition with gradient echo sequence for T1-weighted contrast; R, parallel imaging acceleration factor

^1^Multi-band excitation and reconstruction protocols were kindly provided by the Center for Magnetic Resonance Research in the Department of Radiology of the University of Minnesota, USA.

An automated processing pipeline for brain image analysis and quality control was established for UKB at the University of Oxford’s Wellcome Centre for Integrative Neuroimaging (WIN/FMRIB). This pipeline is primarily based around FSL (FMRIB’s Software Library), and other packages such as FreeSurfer^18,19^. When acquired at the imaging centres, the images are reconstructed from *k*-space on the scanner computer and saved initially as DICOM files. The processing pipeline then converts these files to the NIFTI format and undertakes pre-processing (e.g., correcting for head motion and other artefacts) as well as automated quality control that identifies issue with the equipment (e.g., coil failure) and artefacts specific to the participant or scanning session (e.g., excessive head movement). The NIFTI files for the T1 and T2 scans are the default version provided to researchers, as these are suitably “defaced” to remove the possibility of re-identification of any individual participant. In 99.5% of cases, the defaced mask does not overlap with the brain mask^19^. The pipeline also automatically generates thousands of IDPs, such as regional grey matter volume from T1 scans, volume of white matter hyperintensities from T2 scans, fractional anisotropy measures from dMRI scans and signal changes in response to stimuli from task fMRI scans. These IDPs have been made available to researchers in batched uploads to the resource since the imaging enhancement began. In-depth information on the brain MRI protocol and quality control process have been published elsewhere^18,19^.

### Cardiac MRI

Cardiac MRI captures information related to both the structure and function of the heart and can provide a range of measures which have been implicated in cardiovascular disease such as left ventricular mass, left ventricular ejection fraction, left atrial volume and aortic stiffness^20–23^. Prior to UKB, the largest studies of cardiac MRI were the Multi-Ethnic Study of Atherosclerosis (5000 participants)^2^, the Dallas Heart Study (3000 participants)^24^ and the Jackson Heart Study (2000 participants)^25^. Although these studies are valuable resources for population health research, their main focus is on cardiovascular disease and risk factors. The sheer size of UKB offers unique opportunities to investigate the subclinical cardiovascular mechanisms related to a wide range of cardiovascular and non-cardiovascular diseases, including research to identify early markers of pathology and their genetic and lifestyle determinants.

The cardiac MRI scan is performed using a Siemens 1.5 Tesla MAGNETOM Aera scanner (Siemens Healthineers, Erlangen, Germany) with VD13A software and a spine and body flex matrix coil. No pharmacological stressor or contrast agent is used. The protocol lasts ~20 min and provides structural and functional measures of the left and right ventricles, left and right atria and the aorta, including volumes, changes in volumes during cardiac cycle, cardiac wall thickness and mass, tissue motion using tagging and thoracic aorta size and distensibility. Table 3 reports the selected parameters of the cardiac MRI protocols.

**Table 3:** Cardiac MRI protocol parameters.

Protocol name	Resolution (mm^3^)	Matrix	Other parameters
LAX	1.9x1.9x6	210x208x50	TE/TR = 1.16/32 ms, α = 65°, R = 2
SAX	1.8x1.8x8	210x208x50	TE/TR = 1.1/32 ms, α = 10°, R = 2
shMOLLI	0.9375x0.9375x8	variablex384x7	TE/TR = 1.073/400 ms, α = 35°, R = 2
Aorta	1.58x1.58x6	240x196x100	TE/TR = 1.17/28 ms, α = 66°, R = 2
LVOT	1.9x1.9x6	210x208x50	TE/TR = 1.16/32 ms, α = 65°, R = 2
FLOW	1.77x1.77x6	192x92x30	TE/TR = 2.47/37.12 ms, α = 20°, R = 2
TAGGING	1.38x1.38x8	256x256xvariable	TE/TR = 3.89/40.95 ms, α = 12°

LAX, long-axis view imaging; LVOT, left ventricular outflow tract/aortic valve imaging; R, parallel imaging acceleration factor; SAX, short-axis view imaging; shMOLLI, shortened modified Look-Locker imaging

At present, only a limited range of features are automatically extracted from the cardiac scanner, such as inline ventricular function (which assesses left ventricular contours and volume). A group based at Queen Mary University, London, and the University of Oxford has created a cardiac structural MRI segmentation reference by manual analysis of the first 5000 scans^26,27^. However, the scale of the imaging enhancement has accelerated efforts to develop automated processing tools that can extract a wider range of cardiac phenotypes in order to maximise the scientific utility of these data. A range of algorithms to automatically segment and assess the quality of the remaining cine cardiac MR images are now being made openly available^28–30^. An automated large-scale image quality control, analytics and image-based phenotype extraction has been established in collaboration with the University of Leeds Centre for Computational Imaging & Simulation Technologies in Biomedicine (CISTIB) based on a private deployment of the MULTI-X secure-based platform (www.multi-x.org)^30,31^. In-depth information on the cardiac MRI protocol have been published elsewhere^26,32^.

### Abdominal MRI

Anthropometric measures, such as weight, height, BMI and waist-to-hip ratio, are commonly collected in epidemiological studies and have informed our knowledge about the role of adiposity with disease risk. However, these measures are fairly crude indicators of body composition and provide little information on the type and distribution of body fat, which have been shown to be important predictors of disease risk^33^. For example, visceral obesity (abdominal fat surrounding the internal organs) has been linked to an increased risks of type II diabetes, cardiovascular disease, cancer and mortality^34–36^. Accumulation of ectopic fat in the liver can cause hepatic steatosis (fatty liver), which is associated with insulin resistance and hepatocellular carcinoma^37,38^. MRI is considered the gold standard for body composition measurement and offers an unprecedented opportunity to measure internal and ectopic fat content, as well as whole-body and site-specific fat and muscle volume. However, abdominal MRI has not been conducted in any large-scale studies previously. Consequently, UKB is an unprecedented resource to further our understanding of how body fat composition and distribution influences disease risk.

The abdominal MRI protocol follows the cardiac examination on the 1.5 T scanner, employing relevant elements of spine and body matrix coils. The scan includes sequences that last 10 min. Table 4 reports the selected parameters of the abdominal MRI protocols.

**Table 4:** Body MRI protocol parameters.

Protocol Name	Resolution (mm^3^)	Matrix	Other parameters
Dixon Stage 1	2.232x2.232x3	224x168x64	TE/TR = 2.39, 4.77/6.67 ms, α = 10°
Dixon Stage 2	2.232x2.232x4.5	224x174x44	TE/TR = 2.39, 4.77/6.69 ms, α = 10°
Dixon Stage 3	2.232x2.232x4.5	224x174x44	TE/TR = 2.39, 4.77/6.69 ms, α = 10°
Dixon Stage 4	2.232x2.232x4.5	224x174x44	TE/TR = 2.39, 4.77/6.69 ms, α = 10°
Dixon Stage 5	2.232x2.232x3.5	224x162x72	TE/TR = 2.39, 4.77/6.69 ms, α = 10°
Dixon Stage 6	2.232x2.232x4	224x156x64	TE/TR = 2.39, 4.77/6.69 ms, α = 10°
shMOLLI Liver	1.146x1.146x8	384x288x7	TE/TR = 1.93/480.6 ms, α = 35°, R = 2
LMS	1.719x1.719x10	256x232x6	TE/TR = 1.2 (min) TE/TR = 7.2 (max)/14 ms, α = 5°
VIBE	1.1875x1.1875x1.6	320x260x52	TE/TR = 1.15/3.11 ms, α = 10°, R = 2
shMOLLI Pancreas	1.146x1.146x8	384x288x7	TE/TR = 1.93/480.6 ms, α = 35°, R = 2
ME GRE Pancreas	2.5x2.5x6	160x160x10	TE/TR = 2.38(min) TE/TR = 23.8(max)/27 ms, α = 20°

LMS, LiverMultiScan protocol incorporating abdominal T1-weighted T2* and proton density fat fraction mapping; ME GRE, multi-echo gradient echo imaging; R, parallel imaging acceleration factor; shMOLLI, shortened modified Look-Locker imaging; VIBE, volumetric interpolated breath-hold examination

Localisation is performed relative to the jugular notch, which is the centre position of the first stage of the Dixon imaging. The examination includes the LiverMultiScan protocol, developed by Perspectum Diagnostics (Oxford, UK), which images the liver by a single transverse slice at the porta hepatisis using two different sequences^39^. A single breath-hold cardiac-gated Modified Look-Locker Inversion Recovery sequence (shMOLLI) for T1 mapping is acquired. A single breath-held spoiled-gradient-multi-echo sequence in the same slice position is performed. Together, these allow multiple measures sensitive to liver fibrosis, iron content and fat^39^. For volumetric evaluations of the pancreas, a 3D VIBE is acquired in transverse orientation centred at the position of the pancreas. A shMOLLI sequence is performed using the same parameters as for the liver. Finally, multi-echo sequence is used (10 different echoes) to allow measurements of iron and fat content.

For quality control, images are visually inspected immediately after reconstruction at the scanner. Fully automated tools are not currently available for extracting quantitative parameters from the images. However, research groups are developing semi-automated tools to extract fat, muscle and organ measures, including visceral and ectopic fat content^40^ and liver measures^41^. In-depth information on the abdominal MRI protocol have been published elsewhere^40,42,43^.

### Dual-energy X-ray absorptiometry

DXA captures precise site-specific (e.g. proximal femur, lumbar spine) measures of bone mineral density and whole-body composition (e.g. bone, fat and lean mass), with no extensive additional processing and analysis^44^. DXA is regarded as the ‘gold-standard’ tool for the diagnosis of osteoporosis^45^, and can also provide information concerning the joint and its articular surfaces that is relevant to osteoarthritis^46^. Although several population-based cohorts have performed DXA scanning on several thousand participants^47–49^, UKB will be about 10-times bigger and uniquely offers the opportunity to compare body composition measures across DXA and MRI modalities. It also enables the investigation of how bone and joint integrity measures are related to a broad range of health outcomes and their genetic and environmental determinants.

An iDXA instrument (GE-Lunar, Madison, WI, USA) is used in the imaging enhancement to measure several body sites using a protocol that lasts 20 min. The instrument captures high-resolution images of the whole-body, proximal femur, spine (from L4 to T4) and knees, which can be used to identify joint pathology, vertebral fractures and other phenotypes using advanced techniques^50^. Measures of bone mineral density and body composition are automatically derived from the scanner and are transferred to UKB requiring little post-processing. Other measures are also being derived, including indices of bone strength, such as trabecular bone score (a measure of bone texture) and hip structural analysis, as well as hip and knee osteoarthritis phenotypes. High-resolution images of hips, knees, whole body, anteroposterior lumbar spine and lateral thoracolumbar spine are exported as DICOM files for further analysis by researchers.

All radiographers are trained according to a protocol harmonised across the scanning sites to allow consistent, accurate participant positioning and image acquisition. The DXA instrument undergoes manufacturer’s daily quality control and local calibration using a phantom (GE-Lunar, Madison, WI). Periodic calibration across sites and over time is undertaken using a European spine phantom to ensure consistent measures are obtained^51^. An automated quality control protocol, where specific DXA analysis results (femoral neck bone mineral density, dual femur total bone mineral content, trunk fat mass, age, DXA weight) are checked for consistency, is being developed and a random sample of 50 scans per site are checked each quarter, with further radiographer training recommended as appropriate.

### Carotid ultrasound

Carotid ultrasound imaging provides information about the health of the carotid arteries including measures of vessel thickness (expressed as carotid intima-media thickness (CIMT)) and vessel wall and plaque volume^52^. These measures are useful indicators of vascular pathology, such as atherosclerotic burden, and are predictive of various cardiovascular diseases, such as stroke, myocardial infarction and coronary heart disease^53^.

Participants are imaged using a CardioHealth Station (Panasonic Healthcare Corporation of America, Newark, NJ, USA), which has a 9 MHz linear array transducer. The protocol lasts 10 min. Both right and left carotid arteries are imaged using a 2D sweep along the transverse plane from below the carotid bifurcation to below the jaw and is repeated along the longitudinal plane. The CIMT is measured at predefined angles: 150° and 120° on the right carotid artery, and 210° and 240° on the left. A marker is placed on the screen to guide the operator in aligning the flow divider and a 10 mm region of interest box is overlaid and automatically tracks the far wall of the common carotid. After three consecutive cardiac cycles, the image auto-freezes in end-diastole and records the mean, maximum, and minimum CIMT for each angle of acquisition.

All the CIMT measures are automatically generated by the device and do not require further post-processing. However, the quality of data acquisition depends highly on the operator and hence quality control is a high priority. Trained radiographers complete a manual assessment of image quality for all scans against agreed criteria, based on expected key features of the image and automated CIMT measurement^54^. Although vessel wall volume and plaque volume are not available as automated measures within the CardioHealth station, bespoke analysis tools to extract these measures are in development. In-depth information on the carotid ultrasound protocol and quality control process have been published elsewhere^54^.

### 12-lead electrocardiogram

Participants also undertake a 12-lead ECG assessment (GE Cardiac Acquisition Module CAM-14) during the imaging assessment. ECG can be used to detect abnormalities related to heart rhythm and electrical activity and to make inferences regarding cardiac structure^55^. Both major and minor ECG abnormalities have been associated with an increased risk of coronary heart disease^56^ and cardiovascular-related mortality^57^. In UKB, the ECG is performed with participants at rest on the same couch used for the carotid ultrasound. The leads are placed on the right and left forearms proximal to wrists, right and left lower legs proximal to ankles and chest with the measurement lasting 20 s. After acquisition, a summary page displays the results for the operating staff member to either mark as ‘complete’ or provide a reason for incomplete assessment. The ECG system includes interpretation software (GE CardioSoft system) that provides an automated output for the detection of arrhythmias and metrics reflecting electrical activity, such as PR interval, QRS duration and QT interval. The raw ECG datasets are also made available for research use.

### Characteristics and data completeness for the first 40,000 participants

Table 5 summarises selected demographic and lifestyle characteristics for the participants who completed imaging between 2014 and early 2020. A high proportion have undertaken other UKB data enhancements, with almost half (44%) having worn the 7-day accelerometer, compared with 19% of the whole UKB cohort, and most have completed the web-based questionnaires.

**Table 5:** Characteristics and data completion for the first 48,000 participants who attended the imaging enhancement.

	Imaged participants		Non imaged participants
	At imaging %	At baseline %	At baseline %
Characteristic			
Age, mean (SD)	64 (7.5)	55. (7.6)	56. (8.1)
Females	52	52	55
Professional qualifications	74	73	58
Non-white ethnic background	3	3	6
BMI ≥ 30 kg/m^2^	19	18	25
Current smoker	3	6	11
Completed following enhancements			
Physical activity monitor	44		18
Online questionnaires			
24 h dietary recall at least once	60		32
Cognitive function	54		22
Occupational history	55		21
Mental health	69		27
Digestive health	77		30
Repeat of baseline assessment during 2011–2012 (not imaging)	17		3

*BMI* body mass index, SD standard deviation.

More than 80% of participants who have undertaken imaging have complete ‘core’ datasets for each of the imaging modalities, and over 90% have complete ‘core’ datasets for the DXA and carotid ultrasound (Fig. 1). For the small proportion of participants with incomplete data, approximately half arise from participant specific issues, such as inability to comply with the demands of the protocol; for example, failure to complete the brain MRI because of excessive movement (1%) or a sudden episode of claustrophobia (2.2%). Other reasons for missing data for the brain MRI include scanner failures (2 %), staffing issues (0.4%) or scheduling problems (1.1%).

### Data generation, storage and access

Around 2.7 GB of imaging data are generated per participant, with 500TB of storage estimated to be required for 100,000 participants. Imaging data are transferred from the scanners to the data repository via a custom-built Picture Archiving and Communication System (PACS), at the Nuffield Department of Population Health, University of Oxford. The imaging system was initially set up around a commercial PACS system, but due to the quantity and nature of the imaging workflow, a fully customised solution was essential. Images on the PACS are automatically checked for completeness and then replicated in a core archive. Incomplete datasets are flagged for manual checking and fault resolution. The PACS also has a workflow to manage and track potentially serious incidental findings, enables secure access for radiologists and specialists to view image data, write and review reports, and prepare the correspondence and imaging data for the NHS. All personal identifiers are removed before providing participant data to researchers.

The UKB resource is available to all bona fide researchers who are associated with academic and commercial institutions anywhere in the world^58^. Researchers must first register with UKB and can then apply to access the data for specified research projects via an online Access Management System (www.ukbiobank.ac.uk/register-apply), which consists of a brief application form and the selection of data-fields. Applications can be broad in scope as long as the aims of the project are well-defined and consist of health-related research in the public interest.

UKB became available for researchers to access in 2012, with imaging data for the first 5,000 participants available in mid-2015 and for ~40,000 participants by early 2020. Researchers can request IDPs or the scans if they wish to extract novel features. Example images for each modality are provided on the UKB Data Showcase (http://biobank.ndph.ox.ac.uk/showcase/). Imaging data are uploaded to the resource every 6–12 months in batches of 5,000–10,000 participants, so researchers can update their analyses. Researchers are expected to publish and return their results (i.e., code/syntax, derived variables) so that any imaging-derived phenotypes generated as part of a research project are incorporated back into the UKB resource and are available to others.

### Repeat multi-modal imaging on 10,000 participants

Although imaging 100,000 participants is a unique and powerful enhancement to the UKB resource, these data are currently only collected at a single time point and many valuable insights could be gained from observing change in imaging phenotypes over time. Serial measures are necessary to explore trajectories and progression of pathological processes and several measures collected over time typically provides a more accurate insight than a single, cross-sectional measure. For example, measures of change in structural brain MRI are a much better predictor of conversion from mild cognitive impairment to Alzheimer’s disease compared to a single measure^59^. Using cardiac imaging, left ventricular mass has been shown to decrease with age in men when examined cross-sectionally, but to increase with age when examined longitudinally in the same study, demonstrating the importance of multiple measures^60^. Repeated measures also enable researchers to account for random measurement error and within-person variability, known as regression dilution bias, that can bias observed associations towards the null^61^.

Recognising this, at least 10,000 of the imaged participants will be re-invited to undergo a complete repeat of the imaging enhancement. Invitations to participants who had attended the first imaging assessment at least 2 years previously were initiated in May 2019 for the Central region and July 2019 for the Northern region. Although still in the early stages, the response rate has been high (more than 65%), with ~3200 participants having booked appointments to attend repeat imaging within the first seven months.

### Discussion and future directions

By early 2020, almost 50,000 participants had undertaken the imaging assessment, with 100,000 participants expected to have completed the protocol by the end of 2023. Of these, 10,000 participants are expected to have undergone a repeat of the imaging assessment by 2023. Imaging at such a large scale is unprecedented^1,2^. However, as only a small proportion of individuals will go on to develop certain diseases and the influences of risk factors may be small, a large sample size (i.e., in the order of 100,000) is necessary to adequately detect reliable associations with all but the most common conditions and strongest risk factors. The wealth of phenotypic and genetic data available on the UKB cohort will enable researchers to study how imaging phenotypes are related to a wide range of lifestyle, environmental and genetic factors, and to study how these antecedent factors influence disease risk through changes in tissue structure and/or function.

An advantage of the imaging enhancement is that it is embedded in an existing cohort study that has thousands of researchers worldwide actively working with the data. As of early 2020, over 1750 UKB projects were underway, two-thirds of which had received IDPs, and a quarter had received the scans. Hence, while still in the early stages of data acquisition, the imaging data that has been collected (and which is being made available to researchers in regular tranches) has received widespread interest worldwide and is already being used to address a range of novel research questions.

Published output to date has primarily focused on exploring cross-sectional associations between lifestyle factors with IDPs. For example, higher BMI and waist-to-hip ratio have both been associated with smaller volumes in different regions of the brain^62,63^, while hypertension and other vascular risk factors have been linked with abnormal white matter microstructure and other adverse brain measures^64,65^. These early findings could help us understand the mechanism through which vascular risk factors are related to neurodegenerative diseases, such as Alzheimer’s disease^9^. A range of cardiovascular risk factors have also been associated with cardiac structure and function^66–68^, and other, less obvious, associations have been identified, such as with air pollution^69^, menopausal hormonal therapy^70^ and lung function^71^. Novel findings are already emerging, for example diabetes has been shown to be associated with abnormal morphologies and function in all four heart chambers, whereas previously only the left ventricle was typically thought to be affected by diabetes^72^.

In addition to lifestyle factors, there is great interest in exploring how genetic variation is associated with imaging phenotypes to better assess the genetic determinants of early disease and to help understand the biological mechanisms underlying disease associations. For example, genome-wide association studies on over 3000 functional and structural brain imaging phenotypes on 12,000 participants identified novel associations between genes linked to iron transport and IDPs related to lower cognitive function^73^. Other studies have explored the genetic determinants of regional brain volumes and measures of white matter integrity^74–79^. One study provided evidence that genes associated with left-handedness are linked to cortical regions involved in language^80^. These studies are both novel and powerful as previous genetic studies have tended to focus on a narrow selection of imaging-related outcomes and incorporate data from multiple studies (with heterogeneous data collection and analytic techniques), to achieve a sufficiently large sample size.

The brain IDPs are extracted through a fully automated pipeline (established by WIN/FMRIB) and are therefore relatively easy to integrate into the resource and provide for research use. At present, IDPs from the other imaging modalities are extracted using semi-automated or manual pipelines, which are more time consuming and less readily available. However, the sheer scale of the imaging data available in UKB is facilitating the development of new methods to extract novel IDPs structural measures from the cardiac scans^28,81,82^, body compositional measures from the abdominal MRI^40,43,83^, and measures of fat and iron from the liver MRI^39,42,84^. In accordance with UKB access policies, all results, including individual-level IDPs and the methods used to generate them by research users of the resource, are returned to be integrated into the resource so that they can be made available to everyone approved to use the data.

The imaging enhancement has coincided with recent major advances in applications of machine learning and AI, in particular the development of computational algorithms that can learn how to extract meaningful information from raw images^85,86^. This includes the automated segmentation and classification of anatomical structures as well as the detection of abnormalities. Recently, machine learning techniques such as deep learning have demonstrated enormous potential as diagnostic tools by identifying conditions on a par with experts, for example skin cancer and diabetic retinopathy^29,87^. Development of these algorithms requires training on thousands of images to produce robust results, making UKB an ideal dataset. Machine learning techniques were applied to UKB cardiac MRI scans to identify aortic valve malformations and subsequent major cardiac events^88^. Although this analysis was performed on only the first 10,000 imaged participants, it clearly demonstrates the use of UKB as a valuable resource for AI research using the imaging data.

A key aspect of UKB is its prospective study design, which will  enable research concerning the associations between IDPs and a range of incident health outcomes. This is important, as cross-sectional analyses cannot determine temporality of an observed association and are particularly affected by reverse causation, i.e., when the outcome influences the exposure. Data from national death and cancer registries and hospital inpatient data are available for the full cohort, with data from primary care made available for about half the cohort in late 2019. Primary care will be immensely valuable for capturing conditions often diagnosed outside of a hospital inpatient setting. For example, compared to using only death registry and hospital admissions records, incorporating primary care data could increase the number of incident cases identified in the UKB cohort by 2021 by ~150% for dementia (5400 to 13,000 cases), by ~50% for stroke (8300 to 12,900 cases), by over 100% for chronic obstructive pulmonary disorder (13,300 to 30,600 cases) and by ~100% for Parkinson’s disease (2000–4000 cases). Additional case ascertainment is being aided by online questionnaires which are being developed to collect information on outcomes poorly captured through medical records, such as those related to cognitive function, digestive health, pain, sleep and mental health.

External academic and commercial groups are also enhancing the resource by performing cohort-wide assays on the biological samples, including whole genome sequencing (funded by government, charity and industry), exome sequencing (led by Regeneron and a consortium of industry partners), leukocyte telomere length (University of Leicester, UK) and NMR-metabolomics (Nightingale Health). In line with UKB return of results policy, these data will be returned to UKB and integrated into the resource to be accessible to all researchers registered with an approved application.

## Conclusion

UKB is currently on course to collect detailed, high quality multi-modal imaging data on the brain, the heart, abdominal composition, bones, joints and blood vessels on 100,000 participants. UKB has also begun the process of performing repeat imaging on at least 10,000 participants. The amount of imaging data collected on such a large number of participants is truly unique. Yet it is the combination of these data with the wealth of other phenotypic, genetic and medical record information available in UKB that provides a powerful resource to address previously unanswerable research questions. Traditionally, imaging data might be perceived to be of value mainly to specialists in a narrow range of fields. However, researchers from many disciplines, including, but not limited to, epidemiologists, neuroscientists, statisticians, geneticists and psychologists, can and are using the IDPs already available from the imaging scans to conduct health-related research to provide new insights into the prevention, diagnosis and treatment of disease.
